# A phenomenological–hermeneutic study exploring caring responsibility for a chronically ill, older parent with frailty

**DOI:** 10.1002/nop2.467

**Published:** 2020-03-05

**Authors:** Helle Elisabeth Andersen, Bente Hoeck, Dorthe Susanne Nielsen, Jesper Ryg, Charlotte Delmar

**Affiliations:** ^1^ Department of Public Health Nursing Aarhus University Aarhus Denmark; ^2^ Health Sciences Research Centre UCL Odense Denmark; ^3^ Department of Public Health University of Southern Denmark Odense Denmark; ^4^ Department of Clinical Research University of Southern Denmark Odense Denmark; ^5^ Migrant Health Clinic Odense University Hospital Odense Denmark; ^6^ Department of Geriatric Medicine Odense University Hospital Odense Denmark; ^7^ Institut for Helse‐ og omsorgsfag Norway's Artic University Tromsø Norway; ^8^ VID Helsefag Bergen Oslo Norway

**Keywords:** adult children, caring responsibility, diaries, frailty, illness, interviews, nursing, older parents 80+, phenomenological–hermeneutic, qualitative research

## Abstract

**Aim:**

To provide lifeworld insights into experiences of adult children with caring responsibility for an 80+‐year‐old chronically ill parent with frailty.

**Background:**

Informal care is common in Nordic welfare countries; however, little is known about adult children's experience of caring responsibility in this setting.

**Design:**

A phenomenological–hermeneutic study based on Reflective Lifeworld Research.

**Methods:**

Diaries and semi‐structured interviews with 12 adult children.

**Results:**

Caring responsibility is identified as “a condition of life, filled with uncertainty.” Three constituents contribute to this phenomenon: (a) balancing love, duty and reciprocity; (b) being the parent's advocate and manager; and (c) experiencing concerns and bodily strain.

**Conclusion:**

Adult children work hard to provide care and enhance the well‐being of their parent. Heidegger's concept ‘Fürsorge’ may help us understand how by showing how caring responsibility means balancing different roles vis‐à‐vis the parent, one's own life and the health and social systems. Caring responsibility changes the relationship between parent and child.

## 1 INTRODUCTION

The number of people aged 80 years or more, also called the “oldest‐old,” is increasing worldwide. According to UN projections (UN, 2015:9), the number will have tripled to 434 million by 2050. In Denmark (population total 5.8 million), the number of oldest‐old will have doubled to 500,000 by 2040 (Statistics Denmark, 2018). Caring responsibility for ageing parents is expected in most cultures (Stuifbergen & van Delden, 2011), and informal caregiving is generally well‐researched. However, in Nordic welfare countries, little is known about adult children's experience of caring responsibility for the oldest‐old.

## 2 BACKGROUND

Advanced age is associated with increased risk of illness, frailty and use of healthcare resources (WHO, 2015). In Denmark, most of the oldest‐old resides in the community; of these, 50% are living alone (Statistics Denmark, 2019) and 32% receive home care (DanAge Association, 2017a). Older people living alone are particularly vulnerable, have poor self‐reported health and face everyday life challenges including mobility issues, risk of social isolation and loneliness (Birkeland & Natvig, 2009; Fisher, Baker, Koval, Lishok, & Maisto, 2007; Kharicha et al., 2007; Rolls, Seymour, Froggatt, & Hanratty, 2011; Taube, Jakobsson, Midlöv, & Kristensson, 2016). They are also at increased risk of unplanned hospitalization (Pimouguet, Rizzuto, Lagergren, Fratiglioni, & Xu, 2017).

In Denmark, health and social services are financed by general taxes and therefore free of charge (Danish Ministry of Health, 2017). Home care and home nursing are provided by the municipalities according to individual need to allow people to stay in their own homes as long as possible. Even so, relatives undertake much informal caregiving (Lewinter, 1999; 2003; DanAge Association, 2017b) like in other countries. Approximately 1/3 of the populations in 20 European countries are informal caregivers (family and friends) (Verbakel, Tamlagsrønning, Winstone, Fjær, & Eikemo, 2017), and informal caregiving is more common in Nordic countries than in Central, Eastern and Southern Europe (Verbakel et al., 2017). Demands on relatives like adult children are thus high and expected to increase because of current structural changes to reduce healthcare costs. However, adult children are typically at a stage in their own lives where they face competing demands and must balance work, parenting, spousal relationships, early retirement and other life demands, while simultaneously facing caring responsibility for their older parent(s) (O'Sullivan, 2014).

Informal caregiving is associated with physical and emotional strain, the so‐called caregiver burden (Adelman, Tmanova, Delgado, Dion, & Lachs, 2014; Bastawrous, 2013; Del‐Pino‐Casada, Frías‐Osuna, Palomino‐Moral, & Pancorbo‐Hidalgo, 2011; Ringer, Hazzan, Agarwai, Mutsaers, & Papaioannou, 2017) but may also be experienced as worthwhile and meaningful (Roth, Fredman, & Haley, 2015; Toljamo, Perälä, & Laukkala, 2012).

Previous studies have focused mainly on hospital settings, the discharge process and home care or nursing home settings (Bridges, Flatley, & Meyer, 2010; Ekström et al., 2019; Lewinter, 2003; Rustad, Cronfalk, Furnes, & Dysvik, 2017; Sivertsen, Lawson‐Smith, & Lindhardt, 2018; Søvde, Hovland, Ulleburst, & Råholm, 2019). Caregivers have been conceptualized as “conductors” responsible for maintaining the “rhythm” necessary for an older person's well‐being, whether at home or while hospitalized (Lowson et al., 2013). In a study of how relatives experienced hospitalization of older persons and collaboration with nurses in an acute ward, Lindhardt, Bolmsjö, and Hallberg (2006) found that some relatives entrusted the older person's care into the hands of health professionals and therefore had some respite from caregiving activities; others did not and adopted controlling behaviours, closely monitoring care and treatment.

Family caregivers have often been studied as a homogeneous group (Jarling, Rydström, Ernst‐Bravell, Nyström, & Dalheim‐Englund, 2019; Juntunen et al., 2018; Lindhardt et al., 2006; Moral‐Fernandez, Frías‐Osuna, Moreno‐Cámara, Palomino‐Moral, & Del‐Pino‐Casado, 2018; Ringer et al., 2017). In a meta‐analysis of spouses, adult children and children‐in‐law, spouses reported more depression symptoms and greater financial and physical burden than adult children and children‐in‐law, which was explained mostly by spouses' higher levels of care provision (Pinquart & Sörensen, 2011). However, adult children become primary caregivers if the parent lives alone and they have a history different from that of a spousal relationship. They would have relied on their parent for support and nurturing, but now they must provide support and assistance to their parent. Bastawrous, Gignac, Kapral, and Cameron (2015) investigated factors contributing to adult children caregivers' well‐being, for example duration and type of caregiving (i.e. instrumental or emotional), parent's condition and impairment type (cognitive impairment was associated with greater depression and higher burden than physical impairments) and parent–child relationship quality.

Adult children's motivation for providing informal care has been discussed in terms of pure altruism, reciprocity or family norms (Klimaviciute, Perelman, Pestieau, & Schoenmaeckers, 2017). However, what does it mean to have a caring responsibility in a Nordic welfare context? As healthcare professionals, we must gain a deeper understanding of this group of relatives who offers support across healthcare levels (Bridges et al., 2010; Lowson et al., 2013; Rustad et al., 2017) and are a vital albeit often underestimated resource in the care and well‐being for older people.

## 3 AIM

To provide lifeworld insights into the experiences of adult children with caring responsibility for an 80+‐year‐old chronically ill parent with frailty.

## 4 METHODS

### 4.1 Design

To explore the lived experience of the phenomenon of caring responsibility from adult children's perspectives, we conducted a qualitative study based on Reflective Lifeworld Research (RLR) (Dahlberg & Dahlberg, 2019; Dahlberg, Dahlberg, & Nyström, 2008). RLR works across phenomenological and hermeneutic philosophies and is focused on the lifeworld; the taken‐for granted‐world of experience (Dahlberg & Dahlberg, 2019). RLR aims to illuminate the essential meaning of a phenomenon which requires openness, flexibility and a reflective attitude towards it throughout the entire process. In RLR, this attitude is called “bridling”; it includes awareness of the impact of our pre‐understandings (Dahlberg et al., 2008), for example scientific theories, being healthcare professionals and having (had) older parents ourselves. Thus, the authors continuously reflected on and discussed the evolving process of understanding, patiently waiting for the essential meaning of the phenomenon to show itself during the analysis.

### 4.2 Participants

Participants were 12 adult children (five sons, seven daughters) aged 38–73 years (mean 58 years) whose parents were 81–98 years old (mean 88 years), chronically ill, living alone with frailty and therefore receiving home care support with activities of daily living (ADL), for example personal hygiene, toileting or eating and with instrumental activities of daily living (IADL), for example cleaning and cooking. Their parents also received home nursing mainly focusing on medication. We used the standard frailty definition: “A medical syndrome with multiple causes and contributors that is characterized by diminished strength, diminished endurance and reduced physiologic function that increases vulnerability for developing increased dependency and/or death” (Morley et al., 2013). Parents diagnosed with dementia were excluded.

In collaboration with staff at a geriatric department, the first author selected participants using a purposeful sampling strategy (Holloway & Galvin, 2017) to ensure selection of those currently experiencing the phenomenon of caring responsibility. Participants were included if they were primary caregivers having contact with a parent daily/several times a week; and if they could read, write and speak Danish. Variation regarding sex, age, occupation and the parent's medical condition and care setting appeared during the selection process and thus allowed diverse perspectives to be addressed (Table 1). After consent from the parent, the first author contacted the participant. Preliminary analysis showed that the 12 participants' lifeworld experiences adequately represented the general structure of the phenomenon of caring responsibility.

**TABLE 1:** Characteristics of the participants and their parents

Participant	Age	Occupation	Siblings	Parent	Parent's age	Parent's medical conditions
Son	65	Retired, former nurse/manager	2 sisters	Father	86	Heart disease and prostate cancer
Daughter	58	Factory worker	1 brother abroad	Mother	83	Severe rheumatoid arthritis and osteoporosis
Daughter	66	Retired, former civil economist and dietitian	1 brother	Mother	92	Dizziness and balance problems
Son	62	Social worker	1 brother	Father	93	Chronic obstructive pulmonary disease and prostate problems
Son	60	Manager	1 sister and 1 brother	Mother	93	Osteoporosis, fall problems and gastric ulcer
Daughter	51	Salesperson	2 brothers	Father	93	Chronic obstructive pulmonary disease and heart disease
Son	57	Service engineer	1 brother	Father	86	Chronic obstructive pulmonary disease and prostate problems
Daughter	65	Cleaning assistant	2 sisters	Mother	87	Diabetes, rheumatoid arthritis and fall problems
Daughter	73	Retired, former drugstore worker	None	Father	98	Heart and circulation problems
Son	57	Technical engineer	1 brother	Mother	83	Diabetes and cancer
Daughter	52	Social worker	1 brother abroad	Father	81	Stroke, fall problems and alcohol abuse
Daughter	38	Healthcare assistant	2 brothers	Father	82	Arthritis and cancer

### 4.3 Data collection

Data were generated from January–September 2018 using diaries and interviews. The adult children completed a 2‐week diary from the time of their study inclusion. They could choose between different formats and received an envelope with an audio‐recorder, a notebook and written guidance with open questions like: “Could you please tell about your experiences during your father's/mother's hospital stay? During the discharge process? During home care assistance?” The diary allowed participants to report their experiences shortly after they occurred and undisturbed by the researcher (Clayton & Thorne, 2000). Eight participants completed the diary: two using the audio‐recorder and six using a handwritten format. The diary length varied with most being a couple of pages long (range 1–20 pages). Four participants did not complete the diary due to distress and a lack of time.

The diary was followed up with an in‐depth interview (Dahlberg et al., 2008) by the first author who used an interview guide and diary notes as starting points to further explore the adult children's experiences with caring responsibility. The guide comprised questions expanding on their experiences with caring responsibility, for example: “What is it like to help and support your mother/father? Can you give examples of the caring responsibility you experience? What does helping you parent mean to you?”

The time and location of the 12 interviews were determined at the participants' convenience: two face‐to‐face interviews were conducted in the participants' homes and 10 telephone interviews were conducted in the evenings. Interviews lasted 40–75 min and were recorded. The first author transcribed verbatim the audio‐recorded/handwritten diaries and interviews for textual analysis.

### 4.4 Ethical considerations

Assuming that both the parent and the adult child were in stressful and vulnerable situations, we gave high priority to ethical considerations during the entire process. Oral and written information about the study was given, including an option to withdraw from further participation at any time. Parent and child had time to discuss participation before providing written consent. Confidentiality and anonymity were assured (Nordic Nurses' Federation, 2003). The study was approved by the Danish Data Protection Agency (reference number 2015‐57‐0066).

### 4.5 Data analysis

All data were analysed and discussed with an open, reflective and bridled attitude following the methodological principles of RLR (Dahlberg et al., 2008). Analysis encompassed empirical data from diaries and interviews as a whole, focusing on differences and similarities in descriptions of meanings across data. This approach was cyclic; data were read thoroughly several times to gain an understanding of the overall picture. Thereafter, significant texts, called meaning units, were marked with notes of initial understanding. Related meaning units from diaries and interviews were then gathered in temporary clusters helping the researchers determine the essential structure of meanings. The clusters were then related to each other to find a pattern that described the essential meanings of the phenomenon of caring responsibility followed by descriptions of meanings further constituting the phenomenon. An example of the analysis process is presented in Table 2.

**TABLE 2:** Example of the analysis process

Related meaning units	Temporary clusters	Constituent
“That was the first time I found out that the emergency department was about to send him home. When I finally got through to a doctor, who was extremely aggravated with me, I was forced to tell him exactly how I felt about the whole situation. After my conversation, my father was sent to bed and moved to the geriatric ward”	Ensuring care and treatment	Being the parent's advocate and manager
“However, as I know all of the people who are involved in his case, all the arrangements with individuals and council employees, all these people ring me. Therefore, I feel more like his business manager”	Coordinating with the people involved	

## 5 FINDINGS

The essential structure of the meanings is that the phenomenon of caring responsibility is “a condition of life, filled with uncertainty.” Three closely intertwined constituents contribute to the phenomenon: (a) balancing love, duty and reciprocity; (b) being the parent's advocate and manager; and (c) experiencing concern and bodily strain.

### 5.1 A condition of life, filled with uncertainty

Adult children experience caring responsibility as a condition of life, filled with uncertainty. This state is always present and closely intertwined with affection and obligation and a feeling of giving back some of the help received from the parent earlier in life. It is a condition to be concerned with one's parent's well‐being:

My father is not spoiled. I am giving back a little bit of what he did for me.

Uncertainty relates to the parent's situation with illness, growing frailty and dependency:

It hurts me to see my strong and proud father crumble within a few months and I am worried about what lies ahead.

However, uncertainty also relates to concerns about how to balance caring responsibility and the caregiver role in different healthcare contexts and one's own life. The health and social services do not always deliver the expected care, which increases uncertainty about the parent's situation staying in his/her own home; and this sparks concerns regarding growing old and dependent oneself.

#### 5.1.1 Balancing love, duty and reciprocity

Adult children's feeling of caring responsibility comes from love and gratitude towards their older parents, most of whom have been there for their children and helped them in earlier years; now the children want to reciprocate. However, not all children/siblings seem to assume caring responsibility, which increases the load on those who do. Often one (or two) of the children, living nearby and having a closer relationship with the parent, becomes primary caregiver. The feeling of duty and reciprocity depends on the relationship with the parent and the family's history:

I may always have had a closer relationship with my parent(s) than my little brother. He does not feel so obliged to visit and help our mother.

It is meaningful to provide informal care related to IADL, for example shopping; cleaning; taking the parent to the dentist or hospital; and administer the parent's finances. However, assisting with intimate ADL like personal hygiene or toileting is not perceived as natural. This is experienced as being inordinate for both child and parent; thus, an unspoken agreement exists that such ADL are primarily handled by home care.

Practical tasks are often foregrounded. Care and support are time‐consuming and can sometimes be burdensome, for example when a mother repeatedly calls her son whom she knows will come and help her. Thus, the older parent occasionally expects that the child will provide help when needed.

Some children are uncertain about when to involve their parents and find it difficult to balance dependence against independence and to maintain the parent's autonomy and dignity. Others aim to protect and relieve their parent of worries by withholding information:

I feel my father is very thankful and trusting. He is quite aware of the work I do for him and he is confident in my ability. However, I am sure he is not totally aware of the colossal workload that is required. There are many interactions with individuals who are vital in his healthcare, both on a spoken and written level. If he was told this information, it would be counterproductive, so there is no point telling him.

Gratitude and a trusting relationship seem essential when one or two of the children become primary caregiver(s). They take on the role of communicating and coordinating with siblings, other family members and the health and social systems. However, communication can be a challenge, especially if the relationship between the family members is problematic:

I have started talking to my angry sister again because it is necessary to communicate and coordinate, e.g., who visits him (the parent) and when. One day, I asked her to do something very specific, so she did, but otherwise she did not do anything.

Caring responsibility is also driven by feelings of duty, closely intertwined with love for the parent:

If my brother and I did not help our mother, she could not stay in her apartment. However, I feel my life goes by helping her. It is annoying, but my conscience forbids me to act differently.

The children have true concerns about their parent's well‐being and feel a huge responsibility. They respond to the uncertain situation by supporting and doing things their parent can no longer manage alone, even if this has consequences for their own lives.

#### 5.1.2 Being the parent's advocate and manager

For some children, having caring responsibility means supervising and taking on the role of being the parent's advocate and manager, which involves mediating the best care and treatment irrespective of setting. The health and social systems are appreciated as they relieve the children from some of their worries about their parent's basic needs. However, relief is not necessarily associated with being satisfied with the care and treatment provided. Uncertainty is still there, and sometimes, the children have to argue with doctors to arrange hospital admittance for their older parents and remind nurses of basic care needs:

My father was placed in a chair for 11 hours in the emergency department. Finally, I had to call them: ‘My father needs to be put to bed, he's old and tired and hasn't had his medicine or even eaten yet’. That was the first time I found out they were about to send him home. When I finally got through to a doctor, who was extremely aggravated with me, I was forced to tell him exactly how I felt about the whole situation. After my conversation, my father was offered a bed and moved to the geriatric ward.

For some, the advocate/manager role means being alert and checking on healthcare professionals to mediate and ensure that the parent receives the right care, treatment and medication; like a daughter who refused to take her mother home from the emergency department before the nurse had inspected her mother's urine; and further investigation revealed a kidney infection. Or another daughter who asked a home care assistant to call a nurse because the assistant was about to tube feed her father without knowing the instructions. Children also discover medication errors, especially regarding antibiotics, at discharge:

At least twice my father has been discharged and not received the prescribed antibiotics. The home care nurse did not notice the error before I made her aware of it.

Advocating means participating in meetings with healthcare professionals, for example discussing the parent's need for home care. A son explains how he and his brother have to participate to explain their father's situation. Otherwise, the healthcare professionals that should help him would leave him within 2 min because their father would not admit the discomfort he experienced and would not ask for help. Being the parent's manager implies much coordination and being the one trying to have an overview of the parent's care arrangements:

The love and familiarity in the relationship have not changed. However, as I know all of the people who are involved in his case, all the arrangements with individuals and council employees, all these people call me. Therefore, I feel more like his business manager.

Caring responsibility enhances being proactive on the parent's behalf. However, uncertainty is experienced when balancing between standing firm and simultaneously not being viewed by healthcare professionals as being troublesome when questioning decisions. The children know that the health and social systems are under pressure but prefer more proactive systems and suggest that “*a contact person who is affiliated with the patient would be helpful*.”

#### 5.1.3 Experiencing concern and bodily strain

Although assuming caring responsibility is a condition of life, it is stressful watching an old parent become ill, frail and dependent and this situation is accompanied by constant concern and uncertainty about the parent's well‐being, including a touch of guilt. A daughter explains that when she leaves her father's home, she thinks that she should turn the car and drive back to him. Uncertainty is enforced because the father is living alone with illness and frailty. This feeling is present although her father receives home care several times a day. In general, the children are concerned about their parent's well‐being and basic needs, for example whether the parent is eating and drinking sufficiently and receiving the agreed care by the health and social systems.

Adult children want to be there and help whenever necessary, but it can be difficult to find time, especially when they are still working, in which case it is very important to have a flexible employer, for example, during the parent's hospital stay. A daughter expresses difficulties with her work this way:

I often had to catch up hours at work because I was off a lot during my father's hospital stay.

Some adult children set aside their own needs, like leisure activities. A daughter explains how she used to take language and dance lessons, but her mother now takes all her time and has done so for a couple of years. Even if the adult child is retired, he/she may be caught between the responsibility for his or her parent and the rest of the family. Being the only child or having a sibling abroad increases the burden. A daughter explains, crying, how she feels like being the only person her mother has. She feels like having the overall responsibility, even though her mother is an independent human being. It is quite stressful living with the uncertainty of what lies ahead, and it leads to bodily strain like constant worrying, nervousness, insomnia and forgetfulness. A son explains how he wakes up in the middle of the night thinking about all kind of things regarding his father; and a daughter expresses how her nerves are in tatters because she never knows what will meet her when she opens the door to her mother's house. Another younger daughter with small children describes how she is under extreme pressure and sometimes has problems remembering things. She experiences that home care cannot manage her father's complex care needs. Therefore, it is a relief for her when her father is hospitalized:

When he is at home, there is never peace; there is always something one has to take into consideration. Now my father is in the hospital; this allows me a moment of peace. As soon as I know his condition is worsening, I revert to my stressed stage.

In addition to uncertainty and concerns regarding their older parent, some adult children describe uncertainty and concerns about ageing and becoming dependent themselves because they experience gaps in the health and social systems:

My concerns also apply to my own life. I am not sure I want to grow old because who will take care of me? My son lives far away, lucky me.

This daughter is very sceptical about the Danish health and social systems and the policy of ageing in place.

## 6 COMPREHENSIVE UNDERSTANDING AND DISCUSSION

The phenomenon of caring responsibility for an old parent with frailty and illness is experienced as “a condition of life, filled with uncertainty”. Our findings highlight adult children's deeply rooted will to show caring responsibility for their parent. Even in a Nordic welfare state like Denmark, caring responsibility is experienced as a condition of life. The children could leave all care to the formal health and social systems, but they do not. They want to reciprocate; expressed as wanting to give back some of the help they received from their parent(s). In line with other studies (Lindhardt et al., 2006; Lowson et al., 2013), they act as advocates and managers to protect and ensure their parents' well‐being. Our findings confirm those of Johansson and Sundström (2006), who showed that solidarity between generations, expressed as willingness to help older persons in need, is not superseded by extensive public care. On the contrary, it has gained new ground in the face of public service curtailments. This may be explained by looking closer at the concept of caring.

Caring means to be concerned about and can be understood as the basis for all human relations (Delmar, 2013, 2018; Martinsen, 1993). According to the German philosopher Heidegger (1962, p. 227), caring or “Sorge” in German is a fundamental basis of our being‐in‐the‐world. Heidegger distinguishes between “Besorgen,” meaning our engagement with things, and “Fürsorge,” meaning our engagement with other people. “Fürsorge” is commonly translated as “solicitude,” suggesting care like in “taking care of children” (Heidegger, 1962, p. 157). Life is one's own self‐being, as well as being at the same time with others, for which caring is constitutive. Therefore, a close connection between care, self‐care and solicitude exists that is inherent in adult children's descriptions of caring responsibility as a condition of life. “Fürsorge” may explain motives of love, duty and reciprocity, which are at play between adult children and their parent in the present study. With “Fürsorge,” care manifests in our everyday life in two ways. The first kind of “Fürsorge” is substitutive; the caregiver is putting him or herself in the other person's place for as long as it takes; the caregiver “leaps in” (einspringen) to take over responsibility for a current situation. In the second kind of “Fürsorge,” the caregiver “leaps ahead” (vorausspringen) of the care recipient to show the way towards future possibilities and potentials. Adult children seem to assume and balance “Fürsorge” in both ways in various combinations: when they take over responsibility and become advocates and managers mediating between their older parent, the health and social services and the rest of the family; and when they balance feelings of love, duty and reciprocity enforced by the fact that the parent lives alone and needs support with IADL. “Fürsorge” is an ongoing condition of life. However, in the situation with an old, ill parent, the practical part of “Fürsorge” seems to background togetherness because the child is busy with the new roles as, for example housekeeper, chauffeur, advocate and manager, thus disrupting balanced reciprocity in the relationship. The child must navigate in this new asymmetrical relationship while still being the son/daughter, trying to maintain the parent's autonomy and dignity.

Regarding dignity, it is noteworthy that an unspoken agreement seems to exist between parent and child that intimate ADL should be handled by home care. This is different from studies from other parts of the world, where adult children may do whatever necessary including providing financial support (Abalos, Yasuhiko Saito, Cruz, & G.T. & Booth, H., 2018; Aires et al., 2017; Mendez‐Luck, Kennedy, & Wallace, 2008). However, this mutual agreement is consistent with findings of prior studies by Haberkern and Szydlik (2010) and Suanet, Groenou, and Tilburg (2012), showing that most of the population in Western European countries favours government responsibility in this respect, especially in the Netherlands and the Scandinavian countries. Interestingly, according to Verbakel et al. (2017), informal caregiving is common in Nordic countries and Denmark has the second‐highest prevalence rate (42.8%) of informal caregivers among 20 European countries. This supports the findings in the present study that adult children are working hard to ensure the well‐being of their parent.

Though being a condition of life, showing caring responsibility is accompanied by uncertainty. With Heidegger's word, care means to be concerned, which implies a degree of uncertainty. The whole situation with an old, ill parent with frailty makes adult children face existential, life‐constraining life phenomena (Delmar, 2013, 2018) like powerlessness and despair, causing concern and bodily strain. These findings are in line with previous research where “caregiver burden” was measured mainly through “burden scales” (Adelman et al., 2014; Bastawrous, 2013; Pinquart & Sörensen, 2011; Ringer et al., 2017). However, uncertainty also relates to how to balance growing dependency and maintaining the parent's autonomy and dignity, balancing feelings of love and duty, when to support and participate in important decisions and when to step back, or with Heidegger's words, when to leap in and when to leap ahead. For some children, it may be difficult to detach themselves from the caregiving role and just be “the child.” They may become entrapped, like the daughter who skipped all her leisure activities. Balancing one's own life, including work‐life if not yet retired, is a challenge. A study by Eldh and Carlsson (2010) confirms how middle‐aged adult children expressed that they seemed to work all the time, either as employees or as an informal caregiver, some even decided to retire earlier than planned (Carlsen & Lundberg, 2018).

Uncertainty is constantly present as an existential concern about what lies ahead regarding the parent's illness, frailty and dependency. Uncertainty is sometimes enforced by the perception of the health and social systems' failure to deliver the expected care and treatment; and some children experience uncertainty when mediating between their parent and healthcare professionals. They try to leap ahead and balance obtaining the best care and treatment for their parent with the risk of being viewed as too demanding. In line with previous research (Bridges et al., 2010), adult children would prefer the healthcare professionals to be more proactive and sensitive, which would prevent some uncertainty. Adult children's experiences with caring responsibility for an older parent raise concerns about growing old and becoming dependent, even in a welfare state like Denmark. These concerns should be considered when planning future care and policies for older people and relatives like adult children.

### 6.1 Study limitations

Using the diary method to allow descriptions of experiences shortly after they occurred was a challenge since the amount of data was minimal due to participants' distress and lack of time. The diary method should, therefore, be considered in combination with other methods and was here complemented by in‐depth interviews allowing rich lifeworld descriptions. Furthermore, telephone interviews are often depicted as a less attractive alternative to face‐to‐face interviews because of the absence of visual cues (Holloway & Galvin, 2017). However, in the present study telephone interviews allowed participants to feel relaxed and able to disclose sensitive information (Norvick, 2007) and describe the phenomenon of caring responsibility in terms of feelings of sadness, anger and love.

## 7 CONCLUSIONS

Despite support from the Danish health and social systems with ADL, IADL and home nursing, adult children in this study worked hard to ensure the right care and treatment for their older parent. The phenomenon of caring responsibility is a condition of life; however, it is accompanied by substantial uncertainty due to the parent's illness and frailty and is enforced by the fact that the parent often lives alone. Reflections on care and Heidegger's concept of ‘Fürsorge’ allows a deeper understanding of the phenomenon, showing how caring responsibility means balancing uncertainty while fulfilling different roles concerning the parent, one's own life and the health and social systems, leading to constant concerns and bodily strain. Caring responsibility changes the relationship between parent and child and makes it more asymmetrical, with the child trying to leap in and leap ahead while balancing the parent's autonomy and dignity.

### 7.1 Implications

Nuanced lifeworld descriptions and comprehensive understanding of the complex phenomenon of caring responsibility from the perspectives of adult children caring for an old, frail, chronically ill parent who lives alone have several implications:

- These insider views can enhance empathic understanding and allow a deeper level of care focusing on patient and family.
- Adult children play a vital role in their parent's care and treatment; thus, it should be considered how such a role can be more actively acknowledged and how adult children can be more actively involved in planning arrangements.
- Supporting relatives like adult children in their caregiver role in a more proactive way, it would be beneficial if a contact person be affiliated with the older parent whether he or she was hospitalized or living at home.
- At a policy level, the contributions and responsibilities assumed by adult children should be recognized since they play a crucial role in the policy of ageing in place. Furthermore, their concerns regarding future elderly care should be considered.

## CONFLICT OF INTEREST

The authors declare no conflict of interest.

## AUTHOR CONTRIBUTIONS

All authors: substantial contribution to and agreement on the final version of the manuscript.

## References

[nop2467-bib-0001] Abalos, J. B. , Saito, Y. , Cruz, G. T. & Booth, H. (2018). Who cares? Provision of care and assistance among older persons in the Philippines. Journal of Aging and Health, 30(10), 1536–1555. 10.1177/0898264318799219 30270711

[nop2467-bib-0002] Adelman, R. D. , Tmanova, L. L. , Delgado, D. , Dion, S. , & Lachs, M. S. (2014). Caregiver burden. A clinical review. Journal of the American Medical Association, 311, 1052–1059. 10.1001/jama.2014.304 24618967

[nop2467-bib-0003] Aires, M. , Mocellin, D. , Fengler, F. , Rosset, D. , dos Santos, N. , Machado, D. , … Paskulin, L. (2017). Association between filial responsibility when caring for parents and the caregivers overload. Revista Brasileira de Enfermagem, 70(4), 767–774. 10.1590/0034-7167-2017-0133 28793107

[nop2467-bib-0004] Bastawrous, M. (2013). Caregiver burden – A critical discussion. International Journal of Nursing Studies, 50(3), 431–441. 10.1016/j.ijnurstu.2012.10.005 23131724

[nop2467-bib-0005] Bastawrous, M. , Gignac, M. A. , Kapral, M. K. , & Cameron, J. I. (2015). Factors that contribute to adult children caregivers' well‐being: A scoping review. Health and Social Care in the Community, 23(5), 449–466. 10.1111/hsc.12144 25472851

[nop2467-bib-0006] Birkeland, A. , & Natvig, G. (2009). Coping with ageing and failing health. A qualitative study among elderly living alone. International Journal of Nursing Practice, 15, 257–264. 10.1111/j.1440-172X.2009.01754.x 19703041

[nop2467-bib-0007] Bridges, J. , Flatley, M. , & Meyer, J. (2010). Older people's and relatives' experiences in acute care settings: Systematic review and synthesis of qualitative studies. International Journal of Nursing Studies, 47, 89–107. 10.1016/j.ijnurstu.2009.09.009 19854441

[nop2467-bib-0008] Carlsen, B. , & Lundberg, K. (2018). ‘If it weren’t for me..’: perspectives of family carers of older people receiving professional care. Scandinavian Journal of Caring Sciences, 32, 213–221. 28475236 10.1111/scs.12450

[nop2467-bib-0009] Clayton, A. , & Thorne, T. (2000). Diary data enhancing rigour: Analysis framework and verification tool. Journal of Advanced Nursing, 32(6), 1514–1521. 10.1046/j.1365-2648.2000.01609.x 11136421

[nop2467-bib-0010] Dahlberg, K. , & Dahlberg, H. (2019). Open and reflective lifeworld research – A third way. Qualitative Inquiry, 1–7, 10.1177/107780041983669

[nop2467-bib-0011] Dahlberg, K. , Dahlberg, H. , & Nyström, M. (2008). Reflective lifeworld research. Lund, Sweeden: Studentlitteratur.

[nop2467-bib-0012] DanAge Association . (2017a). Færre ældre over 80 år får hjemmehjælp (Decrease in people aged 80 years or more, who receives home care). Retrieved from https://www.aeldresagen.dk/presse/pressemateriale/nyheder/faerre-aeldre-over-80-aar-faar-hjemmehjaelp-nu-end-tidligere. Accessed 20 September 2019.

[nop2467-bib-0013] DanAge Association (2017b). Undersøgelse om pårørende. Voxmeter for Ældresagen (Study about relatives). Retrieved from https://www.denoffentlige.dk/sites/default/files/suppliers/news/files/aeldresagen-paaroerende-2017-voxmeter.pdf. Accessed 20 September 2019.

[nop2467-bib-0014] Danish Ministry of Health . (2017). Healthcare in Denmark. An overview. Retrieved from https://www.sum.dk/English/~/media/Filer%20-%20Publikationer_i_pdf/2016/Healthcare-in-dk-16-dec/Healthcare-english-V16-dec.ashx. Accessed 20 September 2019.

[nop2467-bib-0015] Delmar, C. (2013). Becoming whole: Kari Martinsen's philosophy of care – Selected concepts and impact on clinical nursing. International Journal of Human Caring, 17(3), 20–28. 10.20467/1091-5710.17.3.20

[nop2467-bib-0016] Delmar, C. (2018). Scandinavian caring sciences InRosa W., Horton‐Deutsch S., & Watson J. (Eds.), A handbook for caring science (pp. 461–474). New York, NY: Springer Publishing Company.

[nop2467-bib-0017] Del‐Pino‐Casada, R. , Frías‐Osuna, A. , Palomino‐Moral, P. A. , & Pancorbo‐Hidalgo, P. L. (2011). Coping and subjective burden in caregivers of older relatives: A quantitative systematic review. Journal of Advanced Nursing, 67(11), 2311–2322. 10.1111/j.1365-2648.2011.05725.x 21658096

[nop2467-bib-0018] Ekström, K. , Spelmans, S. , Ahlström, G. , Nilsen, P. , Alftberg, Å. , Wallerstedt, B. , & Behm, L. (2019). Next of kin's perceptions of the meaning of participation in the care of older persons in nursing homes: A phenomenographic study. Scandinavian Journal of Caring Sciences, 33, 400–408. 10.1111/scs.12636 30604875 PMC7328681

[nop2467-bib-0019] Eldh, A. C. , & Carlsson, E. (2010). Seeking a balance between employment and the care of an ageing parent. Scandinavian Journal of Caring Sciences, 25, 285–293. 10.1111/j.1471-6712.2010.00824.x 20723154

[nop2467-bib-0020] Fisher, G. S. , Baker, A. , Koval, D. , Lishok, C. , & Maisto, E. (2007). A field test of the Cougar Home Safety Assessment (version 2.0) in the homes of older persons living alone. Australian Occupational Therapy Journal, 54, 124–130. 10.1111/j.1440-1630.2006.00604.x

[nop2467-bib-0021] Haberkern, K. , & Szydlik, M. (2010). State care provision, societal opinion and children's care of older parents in 11 European countries. Ageing and Society, 30(2), 299–323. 10.1017/S0144686X09990316

[nop2467-bib-0022] Heidegger, M. (1962). Being and time (Macquarrie, J. & Robinson, E. trans.). Oxford, UK: Blackwell.

[nop2467-bib-0023] Holloway, I. , & Galvin, K. (2017). Qualitative research in nursing and healthcare (4th ed.). Chichester, UK: Wiley & Sons Inc.

[nop2467-bib-0024] Jarling, A. , Rydström, I. , Ernst‐Bravell, M. , Nyström, M. , & Dalheim‐Englund, A. C. (2019). A responsibility that never rests – The life situation of a family caregiver to an older person. Scandinavian Journal of Caring Sciences. 10.1111/scs.12703 31058334

[nop2467-bib-0025] Johansson, L. , & Sundström, G. (2006). Policies and practices in support of family caregivers. Filial obligations redefined in Sweden. Journal of Aging & Social Policy, 18(3–4), 7–26. 10.1300/J031v18n03_02 17135092

[nop2467-bib-0026] Juntunen, K. , Salminen, A. , Törmäkangas, T. , Tillman, P. , Leinonen, K. , & Nikander, R. (2018). Perceived burden among spouse, adult child and parent caregivers. Journal of Advanced Nursing, 74, 2340–2350. 10.1111/jan.13733 29869807

[nop2467-bib-0027] Kharicha, K. , Iliffe, S. , Harari, D. , Swift, C. , Gillmann, G. , & Stuck, A. E. (2007). Health risk appraisal in older people 1: Are older people living alone an ‘at‐risk’ group? British Journal of General Practice, 57, 271–276. 17394729 PMC2043328

[nop2467-bib-0028] Klimaviciute, J. , Perelman, S. , Pestieau, P. , & Schoenmaeckers, J. (2017). Caring for dependent parents: Altruism, exchange or family norm? Journal of Population Economics, 30, 835–873. 10.1007/s00148-017-0635-2

[nop2467-bib-0029] Lewinter, M. (1999). Spreading the burden of gratitude: Elderly between family and state. PhD dissertation, University of Copenhagen, Copenhagen, Denmark.

[nop2467-bib-0030] Lewinter, M. (2003). Reciprocities in caregiving relationships in Danish elder care. Journal of Aging Studies, 17(3), 357–377. 10.1016/S0890-4065(03)00025-2

[nop2467-bib-0031] Lindhardt, T. , Bolmsjö, I. , & Hallberg, I. (2006). Standing guard – Being a relative to a hospitalised, elderly person. Journal of Aging Studies, 20, 133–149. 10.1016/j.jaging.2005.06.001

[nop2467-bib-0032] Lowson, E. , Hanratty, B. , Holmes, L. , Addington‐Hall, J. , Grande, G. , Payne, S. , & Seymour, J. (2013). From ‘conductor’ to ‘second fiddle’: Older adult care recipients' perspectives on transitions in family caring at hospital admission. International Journal of Nursing Studies, 50, 1197–1205. 10.1016/j.ijnurstu.2012.02.005 22385914

[nop2467-bib-0033] Martinsen, K. (1993). Fra Marx til Løgstrup. Om etikk og sanselighet i sykepleien (From Marx to Løgstrup): Oslo, Norway: TANO A/S.

[nop2467-bib-0034] Mendez‐Luck, C. A. , Kennedy, D. P. , & Wallace, S. P. (2008). Concepts of burden in giving care to older relatives: A study of female caregivers in a Mexico City neighborhood. Journal of Cross‐Cultural Gerontology, 23, 265–282. 10.1007/s10823-008-9058-6 18324460 PMC2930188

[nop2467-bib-0035] Moral‐Fernandez, L. , Frías‐Osuna, A. , Moreno‐Cámara, S. , Palomino‐Moral, P. A. , & Del‐Pino‐Casado, R. (2018). The start of Caring for an elderly dependent family member: A qualitative metasynthesis. BMC Geriatrics, 18, 228 10.1186/s12877-018-0922-0 30253750 PMC6157059

[nop2467-bib-0036] Morley, J. E. , Vellas, B. , van Kan, G. A. , Anker, S. D. , Bauer, J. M. , Bernabei, R. , … Walston, J. (2013). Frailty consensus: A call to action. Journal of the American Medical Directors Association, 14(6), 392–397. 10.1016/j.jamda.2013.03.022 23764209 PMC4084863

[nop2467-bib-0037] Nordic Nurses' Federation . (2003). Ethical guidelines for nursing research in the Nordic countries. Retrieved from https://www.scribd.com/doc/137291209/SSNs-etiske-retningslinjer. Accessed 20 September 2019.

[nop2467-bib-0038] Norvick, G. (2007). Is there a bias against telephone interviews in qualitative research? Research in Nursing & Health, 31(4), 391–398. 10.1002/nur.20259 PMC3238794 18203128

[nop2467-bib-0039] O'Sullivan, A. (2014). Pulled from all sides: The sandwich generation at work. Work, 50, 491–494. 10.3233/WOR-141959 25248534

[nop2467-bib-0040] Pimouguet, C. , Rizzuto, D. , Lagergren, M. , Fratiglioni, L. , & Xu, W. (2017). Living alone and unplanned hospitalizations among older adults: A population‐based longitudinal study. European Journal of Public Health, 27(2), 251–256. 10.1093/eurpub/ckw150 28339511

[nop2467-bib-0041] Pinquart, M. , & Sörensen, S. (2011). Spouses, adult children and children‐in‐law as caregivers of older adults: A meta‐analytic comparison. Psychology and Aging, 26(1), 1–14. 10.1037/a0021863 21417538 PMC4449135

[nop2467-bib-0042] Ringer, T. , Hazzan, A. , Agarwai, A. , Mutsaers, A. , & Papaioannou, A. (2017). Relationship between family caregiver burden and physical frailty in older adults without dementia: A systematic review. Systematic Reviews, 6(1), 55 10.1186/s13643-017-0447-1 28292313 PMC5351063

[nop2467-bib-0043] Rolls, L. , Seymour, J. E. , Froggatt, K. A. , & Hanratty, B. (2011). Older people living alone at the end of life in the UK: Research and policy challenges. Palliative Medicine, 25(6), 650-657. 10.1177/0269216310373165 20621946

[nop2467-bib-0044] Roth, D. L. , Fredman, L. , & Haley, W. E. (2015). Informal caregiving and its impact on health: A reappraisal from population‐based studies. The Gerontologist, 55, 309–319. 10.1093/geront/gnu177 26035608 PMC6584119

[nop2467-bib-0045] Rustad, E. C. , Cronfalk, B. S. , Furnes, B. , & Dysvik, E. (2017). Next of kin's experiences of information and responsibility during their older relatives' care transitions from hospital to municipal health care. Journal of Clinical Nursing, 26, 964–974. 10.1111/jocn.13511 27533604

[nop2467-bib-0046] Sivertsen, D. M. , Lawson‐Smith, L. , & Lindhardt, T. (2018). What relatives of older medical patients want us to know – A mixed‐methods study. BMC Nursing, 17(1), 17–32. 10.1186/s12912-018-0304-0 30069163 PMC6064176

[nop2467-bib-0047] Søvde, B. E. , Hovland, G. , Ulleburst, B. , & Råholm, M. B. (2019). Struggling for a dignifying care: Experiences of being next of kin to patients in home health care. Scandinavian Journal of Caring Sciences, 33(2), 409–416. 10.1111/scs.12638 30604881

[nop2467-bib-0048] Statistics Denmark . (2018). Befolkningsfremskrivning (Population projections). Retrieved from https://www.dst.dk/da/Statistik/emner/befolkning-og-valg/befolkning-og-befolkningsfremskrivning/befolkningsfremskrivning. Accessed 20 September 2019.

[nop2467-bib-0049] Statistics Denmark . (2019). Number of persons in the family and age. Retrieved from https://www.statistikbanken.dk/FAM100N. Accessed 20 September 2019

[nop2467-bib-0050] Stuifbergen, M. C. , & Van Delden, J. J. M. (2011). Filial obligations to elderly parents: A duty to care? Medicine, Health Care and Philosophy, 14, 63–71. 10.1007/s11019-010-9290-z PMC3015170 20922568

[nop2467-bib-0051] Suanet, B. , Van Groenou, M. , & Van Tilburg, T. (2012). Informal and formal home‐care use among older adults in Europe: Can cross‐national differences be explained by societal context and composition? Ageing & Society, 32(3), 491-515. 10.1017/s0144686x11000390

[nop2467-bib-0052] Taube, E. , Jakobsson, U. , Midlöv, P. , & Kristensson, J. (2016). Being in a bubble: The experience of loneliness among frail older people. Journal of Advanced Nursing, 72(3), 631–640. 10.1111/jan.12853 26568280

[nop2467-bib-0053] Toljamo, M. , Perälä, M. L. , & Laukkala, H. (2012). Impact of caregiving on Finnish family caregivers. Scandinavian Journal of Caring Sciences, 26, 211–218. 10.1111/j.1471-6712.2011.00919.x 21859434

[nop2467-bib-0054] UN, United Nations, Department of Economic and Social Affairs, Population Division . (2015). World population ageing 2015. Retrieved from http://www.un.org/en/development/desa/population/publications/pdf/ageing/WPA2015_Report.pdf. Accessed 20 September 2019.

[nop2467-bib-0055] Verbakel, E. , Tamlagsrønning, S. , Winstone, L. , Fjær, E. L. , & Eikemo, T. A. (2017). Informal care in Europe: Findings from the European Social Survey (2014) special module on the social determinants of health. European Journal of Public Health, 27(suppl_1), 90–95. 10.1093/eurpub/ckw229 28355645

[nop2467-bib-0056] WHO . (2015). World report on ageing and health. World Health Organization. Retrieved from http://apps.who.int/iris/bitstream/10665/186463/1/9789240694811_eng.pdf. Accessed 20 September 2019.

